# Comparison of Sports Habits and Attitudes in University Students of Physical and Sports Education of Mostaganem (Algeria) and Physical Activity and Sport Sciences of León (Spain)

**DOI:** 10.3389/fpsyg.2020.593322

**Published:** 2021-01-05

**Authors:** Marta Zubiaur, Abdelkader Zitouni, Saray Del Horno

**Affiliations:** ^1^Department of Physical Education and Sport, Faculty of Physical Activity and Sports Sciences, University of Léon, Léon, Spain; ^2^Laboratory of Applied Sciences to Human Movement, Institute of Physical Education and Sport, University of Abdelhamid Ibn Badis—Mostaganem, Mostaganem, Algeria

**Keywords:** healthy habits, motivation, attitudes, physical education students, Algeria, Spain

## Abstract

**Background:**

In their professional practice, teachers can exert a strong influence on students, promoting healthy habits for life through the example of their own lifestyle. The aim of this study was to compare sports habits and attitudes in Physical Activity and Sports Education students at the universities of León (Spain) and Mostaganem (Algeria).

**Methods:**

We administered the “Motivations and Attitudes Toward Physical Activity and Sports” questionnaire (in Spanish MIAFD) to 125 Algerian university students from the Institute for Physical Education and Sport (age: 21.87 ± 2.51) and 122 Spanish university students (age: 22.98 ± 2.36) from the Faculty of Physical Activity and Sport Science.

**Results:**

Chi-square tests showed significant differences (*p* < 0.001) with a large effect size (*Cramer’s V*: 0.650) in perceptions of sport and satisfaction with participation.

**Conclusion:**

The students from Mostaganem and Leon show many similarities, such as the number of female students in physical education and sport is quite small compared to male students. Both consider that universities should improve sports facilities to promote good practice. The practice of sports by our university students is far superior to that of students with other degrees, suggesting that they will set a good example of healthy habits once they enter their profession. Nevertheless, the participation of female Algerian students in sport was lower than that of Spanish students, and students at León showed more intrinsic motivation for participating in sport than their counterparts at Mostaganem.

## Introduction

There is growing awareness in the West that doing sport and physical activity (PA) is important for health. However, people continue to take less exercise than is recommended by World Health Organization [WHO] (2010) and Rhodes et al. (2017). Admission to a university involves lifestyle changes that often prompt a reduction in PA (Alonso-Fernández and García-Soidán, 2010; Pengpid et al., 2015). In a systematic review, Irwin (2004) found that between 30 and 60% of the university population worldwide is insufficiently active, although rates of active participation varied widely between countries due to the strong influence of social and cultural factors on PA participation and promotion in each country (Stahl et al., 2001; Haase et al., 2004).

Regardless of cultural and socioeconomic differences, one of the factors that many studies point to as essential to the promotion and adherence of PA is the motivation of practitioners. Teixeira et al. (2012), in a systematic review of Deci and Ryan’s theory of Self-Determination (1985) and PA, conclude that the most important element for the adherence and maintenance of sports practice is the development of autonomous self-regulation, either due to intrinsic motivation or through autonomous forms of extrinsic motivation, as demonstrated by the literature reviewed in a wide range of samples and situations. Sevil et al. (2018), analyze the differences, both in motivation and in PA practice since Self-Determination theory (Deci and Ryan, 1985), between high school and university students. They found that the university transition not only meant a decrease in practice but also a decrease in intrinsic motivation and an increase in a motivation toward it, stressing the need to implement PA programs that would increase intrinsic motivation during high school and the university stage.

In school and university education alike, PA educators and promoters can contribute to the acquisition and maintenance of healthy habits, including participation in recreational PA among university students (Molina-García et al., 2009; Martínez-Baena et al., 2011). Research has found that in their professional practice, teachers can exert a strong influence on the acquisition of healthy habits for life through the example of their own lifestyle (Mendoza-Núñez et al., 2013). It is therefore important that as future PA and sports professionals in various social spheres, Physical Education (PE) and Sports students show higher PA motivation and participation than other university students. Several studies have shown that PE students habitually present a higher level of PA than that of students taking other university degree courses (Farinola and Bazán, 2012; Práxedes et al., 2016). However, Physical Activity and Sports Science students at the University of León have complained about their reduced level of PA once admitted to the university because of the new demands of study and their new lifestyle (Pérez et al., 2003). This indicates the need for further research to analyze these students’ lifestyles, due to the example they must 1 day set for young people to promote healthy habits in society.

Spain and Algeria present many cultural differences, from their history and religion to their level of economic development and their education and political systems. These factors may affect attitudes to sport and PA habits in PE and sports students.

We analyzed students at two PE, PA, and Sports departments at the universities of Mostaganem (Algeria) and León (Spain). Although numerous studies have examined sport habits and motivations in Spanish university students, very few have specifically analyzed PE and sports students. Furthermore, we found no studies of Algerian university students with this objective. The goal of the present study was to analyze the PA habits and attitudes to sport and PE in university students specializing in PA, sport, or PE and to compare the results obtained for students at two universities in different countries—Algeria and Spain—with distinct cultures.

## Materials and Methods

### Participants

Our sample consisted of 247 university students: 125 (97 men, 28 women; age: 22.98 ± 2.36) from the Institute for Physical Education and Sport at the Abdelhamid Ibn Badis University of Mostaganem (Algeria) and 122 (86 men, 36 women, age: 21.87 ± 2.51) from the Faculty of Physical Activity and Sport Sciences at the University of León (Spain). The Algerian students were distributed in groups of 25 per year (first core year and second and third year of an undergraduate degree in PE, and first and second year of a master’s degree in PE), while the Spanish students were distributed in groups of 31 students each in the first and second years and 30 each in the third and fourth years, respectively, of a degree in PA and Sport Science (from a total of 80 students per year; hence, the sample was considered representative with a 95% confidence level).

### Instruments

We administered the “Motivations and Attitudes Toward Physical Activity and Sports” questionnaire (MIAFD), developed by Pavón (2004) with students at the University of Murcia and subsequently used in several other studies (Pavón and Moreno, 2006, 2008). We selected this questionnaire to analyze the habits, attitudes, and opinions toward sport and PE in students. The instrument consists of two clearly differentiated parts, only the first of which was used in the present study. This contains 27 open and closed questions intended to collect information on the kind of sport respondents engage in, their perceptions of sport and Pe, and their opinion of the facilities and sport activities available at their university. Several of the questions permit multiple responses. The questionnaire also includes sociodemographic questions on gender, age, sports qualifications, and membership of a federation. The instrument has a high level of internal consistency (α de Cronbach: 0.820).

### Procedure

This study was approved by the Ethics Committee at the University of León and was carried out in accordance with the ethical principles for medical research involving human subjects of the World Medical Association [WMA] (2018).

At the University of León, data were collected between December and March in the academic year 2016–2017. The questionnaire was administered in both paper and digital format, by sending an invitation via Google Drive.

At the University of Mostaganem, the questionnaire was translated into Arabic by Professor Abdelkader Zitouni and his team of PhD students in 2017. The translation was subsequently revised by PE and Arabic language teachers at the university and then piloted with 20 students taking different degree courses. The questionnaire was administered in paper format and was completed between October and November 2017.

The participation in the research was voluntary and that no type of economic or similar incentive was used to participate.

### Data Analysis

We performed a descriptive analysis to obtain response percentages, conducted a comparative analysis between universities for certain variables using *Pearson’s X^2^* and determined effect size using *Cramer’s V*. All analyses were performed using SpSS.21, and statistical significance was set at *p* = 0.05.

## Results

### Sociodemographic Data

We found a much lower percentage of female than male students in both university departments, and this difference was more marked, although without reaching significance, at Mostaganem (22.4 women vs. 29.5% at León). As regard age, 73.6% of the Algerian students were aged between 21 and 25, while 71.3% of the Spanish students were aged between 18 and 21. Approximately half of the students were federated and held a sports qualification (53.6 and 52.8% of Algerian students and 59.3 and 46.7% of Spanish students, respectively).

### Physical Activity and Sport Participation Characteristics

The responses to items concerning participation in sport showed that 100% of the students enjoyed sport, and most, but not all, presented very high percentages of participation (88.7 and 89.5% of male students at Mostaganem and León, respectively, and 88.9% of female students at León, but only 57.14% of female students at Mostaganem). Table 1 shows that the Algerian students participated in fewer specialist sports than the Spanish students, and that football was the most popular sport in both cases and sexes, although differences were observed in all sports except swimming.

**TABLE 1:** Participation in recreational sports by university (*X*^2^ and effect size).

**Sport**	**Mostaganem**	**León**	***X*^2^**	***Cramer’s V***	***p***
Football	49.5%	31.5%	66.489	0.564***	0.000
Handball	8.9%	2.8%			
Volleyball	8.9%	0%			
Basketball	10.9%	6.5%			
Athletics	0%	4.6%			
Swimming	2.0%	2.8%			
Martial arts	3%	7.4%			
Running	0%	5.6%			
Fitness	16.8%	7.4%			
Futsal	0%	6.5%			
Cycling	0%	4.6%			
Triathlon	0%	4.6%			
Other	0%	15.7%			

***p < 0.001; **p < 0.01; *p < 0.05.

Most respondents reported that their level of skill was advanced (76.8% Mostaganem, 71.6% León), although more Algerian than Spanish students considered themselves beginners (18.8 vs. 11.9%), while a higher percentage of Spanish students considered themselves experts (16.5 vs. 4.5%; X2: 9.581, *p*: 0.008). At Monstaganem, Friday followed by Sunday were the most popular days for doing sport, while Saturday was the least popular day. Meanwhile, at León, Sunday was the least popular day, with few differences between the other days. The most popular times were between 20 and 22 h at León and between 15 and 17 h at Mostaganem.

Sport was the favorite recreational activity (70.2% at Mostaganem and 65.6% at León), with differences observed for personal activities, more frequently chosen by the Algerian students (24.8 vs. 9.80%), and social activities, more popular with the Spanish students (15.6 vs. 0%) (*X*^2^: 28.333, *Cramer’s V*: 0.341, *p*: 0.000). We also found significant differences in the form of participation and in the type and use of sports facilities (Table 2).

**TABLE 2:** Form of participation and facilities used, by university.

	**Mostaganem**	**León**	***X*^2^**	**Cramer’s V**	***p***
**Form of participation**					
On my own initiative, alone	35.9%	41.8%	0.777	0.060	0.402
On my own initiative, with friends	17.5%	45.5%	19.850	0.305***	0.000
As an activity organized by the university	19.4%	20.0%	0.011	0.007	0.527
As an activity organized by a federated club	23.3%	56.4%	24.154	0.337***	0.000
As an activity organized by a gym	34.0%	10.0%	18.066	0.291***	0.000
**Facilities used**					
University	64.1%	36.4%	16.343	0.277***	0.000
Private club/gym	7.8%	50.0%	45.549	0.462***	0.000
School/high school	12.6%	2.70%	7.495	0.188**	0.006
Regional council premises	12.6%	35.5%	15.028	0.266***	0.000
Public place (e.g., street, park)	9.7%	42.7%	29.589	0.373***	0.000
Home	57.3%	20.9%	29.721	0.374***	0.000

***p < 0.001; **p < 0.01; *p < 0.05.

### Opinion About Participation in Physical Activity, Sport, and Physical Education

We observed differences between all responses to the question on what gave the greatest satisfaction from doing sport, except for the response, “the support of my parents.” We also detected differences in relation to self-perceptions of skill (Table 3).

**TABLE 3:** Causes of satisfaction from doing sport and self-perceptions of skill.

	**Mostaganem**	**León**	***X*^2^**	**Cramer’s V**	***p***
**Causes of satisfaction from doing sport**					
Playing better than on previous occasions	31.10%	58.90%	15.796	0.272***	0.000
Proving that I am better than others of my age or on my team	53.40%	19.10%	27.280	0.358***	0.000
Health reasons	25.2%	41.8%	6.531	0.175*	0.011
Pleasure of participation in itself	18.40%	65.50%	46.328	0.466***	0.000
Physical exertion	10.70%	48.20%	35.592	0.409***	0.000
The excitement of competition	53.90%	27.30%	15.648	0.272***	0.000
Feeling part of a team and being with friends	26.20%	44.50%	7.789	0.191**	0.006
The support and encouragement of my parents	23.30%	17.30%	1.200	0.075	0.273
**Self-perceptions of skill**					
I’m not very good	3.20%	1.60%	102.906	0.647***	0.000
I’m competent	33.10%	47.50%			
Others think that I’m effective	63.70%	9.80%			
I’m highly skilled	0.00%	41.00%			

***p < 0.001; **p < 0.01; *p < 0.05.

We also found considerable differences regarding their perceptions of sport, starting with the higher number of responses given by the Spanish students, with very high percentages for all the opinions (Table 4). Differences also emerged between the universities in relation to students’ perceptions of the PE classes they had received at school.

**TABLE 4:** Perceptions of sport and Physical Education (PE) classes, by university.

	**Mostaganem**	**León**	***X*^2^**	**Cramer’s V**	***p***
**Perception of sport**					
Sport is healthy	21.80%	86.90%	104.977	0.653***	0.000
Sport relieves stress	12.10%	80.30%	115.279	0.685***	0.000
Sport allows you to interact with others	26.60%	79.70%	68.227	0.531***	0.000
Sport keeps you in shape	20.20%	79.50%	86.643	0.593***	0.000
Sport is an important aspect of our education	41.10%	81.80%	42.709	0.418***	0.000
Sport allows me to test my physical capabilities	28.20%	64.50%	32.361	0.363***	0.000
Sport is of no benefit	14.50%	0.00%	19.108	0.297***	0.000
**Perception of PE classes**					
They were easy	33.90%	59.00%	15.637	0.252***	0.000
They were motivating	36.30%	41%	0.571	0.048	0.450
They were useful	61.30%	39.30%	11.848	0.219**	0.001
The teacher encouraged us to do sport outside school hours	4.00%	31.10%	31.346	0.357***	0.000
PE is more important than any other subject	28.20%	31.10%	0.2521	0.032	0.616
The number of classes was sufficient	2.40%	11.50%	7.840	0.179**	0.005

***p < 0.001; **p < 0.01; *p < 0.05.

We found that 100% of the Spanish students thought that sport and PE should play an important role in life, in contrast to 37.9% of the Algerian students.

### Sport Activities Available at the University

We analyzed various aspects related to this question: first, whether the universities encouraged sport and if this leads to a change in sport or PA and, second, their sports facilities. In all cases, we observed significant differences (Table 5).

**TABLE 5:** Questions regarding whether the universities encouraged sport and subjects’ opinion of the university facilities.

	**Mostaganem**	**León**	***X*^2^**	**Cramer’s V**	***p***
**The university encourages participation in PA and sport**					
Yes	42.7%	65.6%	12.908	0.229***	0.000
No	57.3%	34.4%			
**This has led to a change in sport or PA**					
Yes	21.8%	38.1%	6.565	0.178*	0.012
No	78.2%	61.9%			
**The university has sufficient sports facilities**					
Yes	22.6%	35.5%	4.995	0.143*	0.034
No	77.4%	64.5%			
**What do you think should be built or improved (if you answered no)**					
Outdoor facilities	5.6%	2.5%	97.797	0.692***	0.000
Indoor facilities	23.4%	33.8%			
Indoor swimming pools	71%	13.8%			
All the above	0%	50%			
**The university has good quality sports facilities**					
Yes	19.4%	48.8%	23.639	0.311*	0.034
No	80.6%	51.2%			

***p < 0.001; **p < 0.01; *p < 0.05.

## Discussion

Our goal in the present study was to compare the characteristics of PA and sport participation in students studying physical activity and sport at the universities of Mostaganem and León, and to analyze their perceptions of sport and PE.

Notable among the respondents’ characteristics was the equally low proportion of female students at both universities. This similarity is surprising given the late incorporation of Algerian women into the field of sport and the regression in this respect that occurred in the 1990s (Yahiaoui, 2006, 2013), whereas in Spain, clear progress has been achieved in recent decades regarding the importance given to women’s sport and women’s participation in sports (García-Ferrando and Llopis, 2017). However, the percentage of female students taking this degree at the University of León has not increased since 1987, when it was first introduced; the same is the case for most Spanish universities. In general, female teenagers are unenthusiastic about PE and sports, and consequently, fewer women choose to study related degree courses (Moreno et al., 2006; Pavón and Moreno, 2008; Práxedes et al., 2016).

As regard age, this was very similar at both universities, and approximately half the students were federated and held a sport qualification, which differentiated them from students taking other degree courses (Castillo and Giménez, 2011; Práxedes et al., 2016).

In relation to the characteristics of participation in sport, 100% of the students liked sport, as was to be expected given the degree courses they were studying, and a very high percentage of students did sports, with the exception of the female Algerian students, indicating that women’s participation in sport at Mostaganem remains well below that of the men. As a result of a return to more traditional social practices in Algeria in the 1990s, young women stopped attending PE classes because they feared being stigmatized in a more repressive social climate. Subsequently, following the educational reform of 2003, they have gradually resumed attendance of PE classes but often do not participate in activities (Yahiaoui, 2013) since prevailing of the conservative vision has generated gender stereotypes that directly affect sport and PE (Hariti, 2013).

Despite everything, the female students at Mostaganem still did more sport than most university students from Spain and other countries. Therefore, our subjects differed from those analyzed in studies of other degree courses, which obtained much lower percentages of participation (Castillo and Giménez, 2011; Castañeda and Campos-Mesa, 2012), and we did not observe the typical trend in Spain and other countries toward a reduction in participation in sport on admission to a university (Haase et al., 2004; Molina-García et al., 2009; Romaguera et al., 2011; Sevil et al., 2018).

Although the Spanish sample participated in a wider variety of sports, students of both sexes at both universities chose football as the most popular sport, in contrast to students taking other degree courses (Pavón and Moreno, 2008). Note that in a study conducted in 1990, athletics was the most popular sport among PA and sports students at León, whereas they rarely played football (Márquez-Rosa and Zubiaur, 1990). In Algeria, football has assumed an important cultural and political role, and in the 1980s, football stadiums became sites for displaying ideological and cultural symbols (Amara, 2012).

Students at both universities preferred sport over cultural activities as their main recreational pastime. This is in agreement, although with lower percentages in relation to sport, with the results obtained by Pavón and Moreno (2008) for students at Murcia; furthermore, unlike university students taking other degree courses, a high percentage of those who consider themselves beginners (Pavón and Moreno, 2006, 2008), our subjects at both universities unsurprisingly considered their level of skill to be advanced. However, we observed differences between them as regard their preferred time of day—later in Spain—and least preferred days of the week (Mostaganem-Saturday, León-Sunday) for doing sport, probably due to the different customs of each country. These schedules of Spanish students are in line with other works where it is observed that at the end of adolescence, the athletes show a greater tendency to the evening by the exigencies of the university studies (García-Naveira et al., 2015).

With regard to the form of participation, activities organized by federated clubs predominated among the Spanish students, whereas the Algerian students mainly participated in activities they organized themselves or that were organized by a gym. We also detected differences as regard the facilities used; outdoor facilities were used more in Mostaganem than in León, which is to be expected given the climatic characteristics of the two cities (León is much colder, with long winters), and the Algerian students primarily did sport at the university or at home, whereas the Spanish students used a wider range of facilities (gyms, clubs, parks, streets, and university or council premises). These percentages are very similar to those reported in other studies of Spanish university students taking a variety of degree courses (Pavón and Moreno, 2006, 2008).

In relation to the causes of satisfaction from doing sport, extrinsic motivation appeared stronger among the Algerian students, in the sense that participation was dictated by rewards or stimuli external to the activity itself (satisfaction produced by competition; proving one’s superiority), whereas intrinsic motivation played a greater role among the Spanish students, in the sense that they obtained satisfaction from participation in itself and the stimulus it provides (satisfaction with the pleasure obtained from participation, improving one’s skills and being with friends), in line with the theory of self-determination described by Deci and Ryan (2000). We obtained similar results in relation to students’ perceptions of sport: the Spanish students attributed a higher number of values to sport than the Algerian students, and these values were of a more intrinsic nature. Similar results were obtained by León et al. (2020) with students of Infant Education of the University of Castilla la Mancha.

With regard to PE classes, we found that a moderate percentage of students at both universities considered them motivating and more important than classes in any other subject. The Algerian students considered them more useful, whereas the Spanish students considered them easier, and in both cases—although to a greater extent at Mostaganem—a considerable majority felt they were insufficient. These results differ from those obtained for students taking other degree courses, who have reported that PE classes were difficult, useless, and sufficient (Pavón and Moreno, 2008). It is relevant here that our subjects had chosen to study these degree courses, and therefore, it is highly likely that they had assessed these classes more positively than their classmates when still at school. A notably small percentage of the Algerian students reported that their teachers had encouraged them to do sport outside school hours. Yahiaoui (2013) conducted a retrospective study of PE and school sports in Algeria and reported a concerted policy in the 1970s to incorporate PE into the curriculum, based on modern teaching methods, followed by a political change in 1979, which led to considerable regression in Algerian education with long-term effects. It is therefore not surprising that even students who have chosen to study these degree courses gave a somewhat negative assessment of the classes received.

An analysis of the universities’ sports activities revealed that although the University of León encouraged sport more than the University of Mostaganem, this had not prompted a change in students’ sports habits, and furthermore, many of the students considered its facilities inadequate and of poor quality as they were old and had little equipment. Meanwhile, the Algerian students considered the facilities at the University of Mostaganem even more inadequate and of poor quality, mainly due to the lack of safety. According to Yahiaoui (2013), sport facility infrastructures in Algeria are poor at all educational levels and rarely meet official standards.

Participation in sport at other Spanish universities is often quite low, as is the case at Alicante (Cambronero et al., 2015) and the Galician universities, where many of the students are unaware of the facilities available (Alonso-Fernández and García-Soidán, 2010). At other universities, participation remains low even when the students consider the facilities to be of good quality (Castañeda and Campos-Mesa, 2012; Corbí et al., 2019). It is possible that our subjects were more demanding with regard to facilities since they were more involved and experienced in sport.

Nevertheless, universities, in general, should give greater importance to good maintenance of sports facilities in order to avert deterioration and should organize attractive sport activities that motivate their students and promote sport associations, university team membership, and the organization of sport competitions, as essential aspects to enhance participation in sport at a university (Molina-García et al., 2009).

## Conclusion

An analysis of PE and sport science students at two universities in different countries—Algeria and Spain—with distinct cultures and socioeconomic levels revealed some similarities in participation in sport and several differences in perceptions of sport and the reasons for participation. The percentage of female students was similarly low at both universities, and female Algerian students participated less in sport than the other students. At both universities, recreational participation in sport was considerably higher than in students taking other degree courses, at least in Spain, and football was the preferred sport. Both Mostaganem and Leon students consider that universities should improve sport facilities to promote good practice, and outdoor facilities and practice are more common among Algerian students. Perceptions of sport and reasons for satisfaction from participation were more intrinsic in the Spanish students and more extrinsic in the Algerian students, and the Spanish students attributed a higher number of values to sport. In sum, if these students at the universities of Mostaganem and León maintain their current level of participation once engaged in professional practice, they will provide a good example of healthy, active lifestyle habits for their fellow students and other sport users. In addition, universities must improve their facilities to promote this good practice.

### Study Limitations and Future Lines of Research

Our study has a series of limitations: it would have been interesting to carry out a comparative analysis of the variables age and sex of the participants, as well as to obtain data on the level of practice, the hours of training, and the age of initiation into the sport, in order to better know and compare the students of both countries. We are also aware that we have only analyzed one center in each country and that it is not possible to generalize for all Algerian and Spanish students. In this sense, it would be interesting to be able to extend the sample to other universities as well as to compare with students from other degrees.

Another aspect to point out is the possibility of analyzing and comparing the sports practice of students from different degrees in order to confirm if our subjects really practice more sports and PA than the rest of the university students.

## Data Availability Statement

The raw data supporting the conclusions of this article will be made available by the authors, without undue reservation.

## Ethics Statement

The studies involving human participants were reviewed and approved by the ethics committee of University of Leon. The patients/participants provided their written informed consent to participate in this study.

## Author Contributions

MZ: direction of work, article production, and statistics. AZ: elaboration of the study in Algeria. SD: elaboration of the study in Spain. All authors contributed to the article and approved the submitted version.

## Conflict of Interest

The authors declare that the research was conducted in the absence of any commercial or financial relationships that could be construed as a potential conflict of interest.
